# Optimal strategy in managing advanced melanoma

**DOI:** 10.1111/1346-8138.17068

**Published:** 2023-12-12

**Authors:** Hiroshi Uchi

**Affiliations:** ^1^ Department of Dermato‐Oncology National Hospital Organization Kyushu Cancer Center Fukuoka Japan

**Keywords:** acral melanoma, advanced melanoma, BRAF and MEK inhibitors, immune checkpoint inhibitors, mucosal melanoma

## Abstract

The advent of immune checkpoint inhibitors and combination therapy with BRAF inhibitors and MEK inhibitors has dramatically improved the prognosis of advanced melanoma. However, since acral melanoma and mucosal melanoma, which are rare in Western countries but are major subtypes of melanoma in East Asia, including Japan, have a low frequency of BRAF mutations, there are currently no treatment options other than immune checkpoint inhibitors in most such cases. Furthermore, owing to a lower tumor mutation burden, immune checkpoint inhibitors are less effective in acral and mucosal melanoma than in cutaneous melanoma. The aim of this review was to summarize the current status and future prospects for the treatment of advanced melanoma, comparing cutaneous melanoma, acral melanoma, and mucosal melanoma.

## 1 INTRODUCTION

Melanoma is known as a highly immunogenic tumor because of the frequent occurrence of vitiligo and spontaneous regression of the primary tumor.^1^ Although numerous therapies to induce tumor‐specific immune responses, such as peptide therapy, dendritic cell therapy, and tumor‐infiltrating lymphocyte therapy, as well as therapies to enhance nonspecific immune responses with interleukin 2 and interferons, have been attempted, no immunotherapy had been proven effective in phase 3 trials until the advent of ipilimumab, an anti‐cytotoxic T lymphocyte‐associated antigen 4 (CTLA‐4) antibody (Ab).^2^, ^3^ Molecules that suppress immune responses against self or excessive immune responses, such as CTLA‐4 and programmed cell death 1 (PD‐1), are called immune checkpoint molecules, and malignant neoplasms utilize immune suppression mechanisms mediated by these molecules to escape the host's immune surveillance mechanisms.^4^ Drugs that activate cellular immunity against tumors by blocking inhibitory signals mediated by immune checkpoint molecules are called immune checkpoint inhibitors (ICIs). In addition to anti‐CTLA‐4 and anti‐PD‐1 Abs, anti‐programmed death ligand 1 (PD‐L1) and anti‐lymphocyte‐activation gene 3 (LAG3) Abs have been used in clinical practice based on the results of randomized controlled trials (RCTs) (Table 1). Whole‐exome sequencing has shown that melanoma is one of the most frequently somatic mutated malignancies.^5^, ^6^ The tumor mutation burden (TMB) of melanoma is dependent on ultraviolet light (UV) exposure and exhibits the following order: head and neck region (chronic sun‐exposed areas) > trunk, and upper and lower limbs (intermittently sun‐exposed areas) > sun‐protected areas.^7^ In addition, several clinical trials have shown that the higher the TMB, the more effective the ICIs, and TMB is therefore considered to be a potential biomarker for predicting the efficacy of ICIs.^8^, ^9^, ^10^

**Table 1:** Representative RCTs of ICIs and target therapies.

Phase	Year	Study	Intervention	Control	Line	ORR (%)		Median PFS (months)		Median OS (months)		References
						Intervention	Control	Intervention	Control	Intervention	Control	
3	2010	MDX010‐20	Ipilimumab	gp100	2+	10.9	1.5	2.9	2.8	10.1	6.4	2
3	2011	CA184‐024	Ipilimumab/DTIC	DTIC	1	15.2	10.3	<3	<3	11.2	9.1	3
3	2015	CheckMate 037	Nivolumab	ICC	2, 3	27	10	3.1	3.7	15.7	14.4	34
3	2015	CheckMate 066	Nivolumab	DTIC	1	42	14	5.1	2.2	37.3	11.2	31
2	2015	KEYNOTE‐002	Pembrolizumab (2 mg/kg) Pembrolizumab (10 mg/kg)	ICC	2+	22 28	4	5.4 5.8	3.6	13.4 14.7	10.9	35
3	2015	KEYNOTE‐006	Pembrolizumab (q2w) Pembrolizumab (q3w)	Ipilimumab	1, 2	33.7 32.9	11.9	8.4	3.4	32.7	15.9	33, 37
3	2015	CheckMate 067	niv/ipi nivolumab	Ipilimumab	1	58 45	19	11.5 6.9	2.9	72.1 36.9	19.9	36, 41
3b/4	2019	CheckMate 511	niv3/Ipi1	niv1/ipi3		45.6	50.6	9.9	8.9	NR	NR	42
3	2011	BRIM‐3	Vemurafenib	DTIC	1	48	5	5.3	1.6	13.6	9.7	53
3	2012	BREAK‐3	Dabrafenib	DTIC	1, 2	50	7	5.1	2.7	18.2	15.6	54
3	2012	METRIC	Trametinib	ICC	1+	47	9	4.8	1.5	(−)	(−)	55
3	2014	coBRIM	vem/cobi	Vemurafenib	1	70	50	12.6	7.2	22.3	17.4	56, 60
3	2014	COMBI‐d	dab/tra	Dabrafenib	1	68	55	11	8.8	25.1	18.7	57
3	2015	COMBI‐v	dab/tra	Vemurafenib	1+	64	51	11.4	7.3			58
3	2018	COLUMBUS	enco/bini encorafenib	Vemurafenib	1+	64.1 51.5	40.8	14.9 9.6	7.3	33.6 23.5	16.9	59, 62
3	2020	IMspire150	Atezolizumab+vem/cobi	vem/cobi	1	67	65	15.1	10.6	39	25.8	63, 64
3	2022	COMBI‐i	Spartalizumab+dab/tra	dab/tra	1	68.5	64.2	16.2	12	NR	NR	65
2/3	2022	RELATIVITY‐047	Relatlimab/niv	Nivolumab	1	43.1	32.6	10.2	4.6	NR	34.1	48

Abbreviations: enco/bini, encorafenib plus binimetinib; dab/tra, dabrafenib plus trametinib; DTIC, dacarbazine; gp100, glycoprotein 100; ICC, investigator‐choice chemotherapy; ICI, immune checkpoint inhibitor; niv1/ipi3, nivolumab (1 mg/kg) and ipilimumab (3 mg/kg); niv3/ipi1, nivolumab (3 mg/kg) plus ipilimumab (1 mg/kg) for four doses followed by nivolumab (3 mg/kg); NR, not reached; ORR, overall response rate; OS, overall survival; PFS, progression‐free survival; q2w, once every two weeks; q3w, once every three weeks; RCT, randomized controlled trial; vem/cobi, vemurafenib plus cobimetinib.

Under the 2018 World Health Organization classification, melanoma is classified as melanomas arising in sun‐exposed skin (low cumulative sun damage [CSD] melanoma/superficial spreading melanoma, high‐CSD melanoma/lentigo maligna melanoma, and desmoplastic melanoma) and melanomas arising at sun‐shielded sites or without known etiological associations with UV radiation exposure (such as acral melanoma [AM], mucosal melanoma [MM], and uveal melanoma).^11^ In this review, low‐CSD melanoma and high‐CSD melanoma are collectively referred to as cutaneous melanoma (CM), and desmoplastic melanoma and uveal melanoma are not discussed. Those of East Asian ethnicity, including the Japanese, have a very low incidence of melanoma, less than 1/10th of that of Caucasians,^12^ and the frequency of each melanoma subtype is known to differ greatly between East Asians and Caucasians. In Caucasians, most cases occur on skin affected by UV light, whereas AM, which occurs on the palms, soles, and under the nails, and MM are rare.^13^ In contrast, AM and MM are the major subtypes of melanoma in the Japanese, accounting for about 40% and 15% of the total, respectively.^14^, ^15^ CM, AM, and MM differ considerably at the gene level as well: whole‐genome sequencing analysis revealed that single‐nucleotide variants (SNVs) and insertions/deletions are more common in CM, while structural rearrangements are more common in AM and MM.^16^, ^17^ Since frameshifts caused by SNVs and insertions/deletions are likely to induce peptides that can be neoantigens, CM is more likely to respond to immunotherapy than AM and MM.^18^ In addition, when melanomas are classified into BRAF‐mutant, RAS‐mutant, NF1‐loss, and triple wild type,^19^ about half of CM cases have BRAF mutations, while only 10%–20% of AM and MM cases have them.^17^, ^20^ On the other hand, triple wild type is found in less than 10% of CM cases, but accounts for 30%–40% in AM and MM.^17^, ^21^, ^22^ Mutations in the KIT gene are found in 10%–20% of AM and MM cases.^21^, ^22^ Currently, BRAF and MEK inhibitors are the only molecular‐targeted therapies in use as standard therapy, and although some efficacy has been observed in clinical trials targeting NRAS and KIT,^23^, ^24^ no drug has demonstrated a significant survival benefit, and future development is awaited.

## 2 DEVELOPMENT OF ICIs AND THEIR IMPACT IN REAL‐WORLD CLINICAL PRACTICE

Anti‐cytotoxic T lymphocyte‐associated antigen 4 (CTLA‐4) antibodies (Abs) block the interaction between CTLA‐4 expressed by cytotoxic T cells (CTLs) and co‐stimulatory molecules expressed by antigen‐presenting cells (APCs), thereby blocking inhibitory signals and maintaining CTL activation.^25^ In the first clinical trial of repeated doses of ipilimumab, reported in 2003, three of 14 patients responded and nine had severe adverse events, including colitis, hepatitis, and hypophysitis, the recovery of all of which was achieved with corticosteroids or hormone replacement.^26^ Following the results of large phase 3 trials (MDX010‐20^2^ and CA184‐024^3^), the approval of ipilimumab in the USA in 2011 and in the EU in 2012 marked a major turning point in the treatment of advanced or unresectable melanoma. Although the overall response rate (ORR) of ipilimumab is not high, at about 15% for first‐line treatment^3^ and 10% for second‐line treatment,^2^ it has characteristics that are critically different from those of conventional cytotoxic anticancer drugs, in that long‐term survival can be expected in responders. A pooled analysis of approximately 5000 cases from prospective trials and expanded access programs showed 3‐year overall survival (OS) of 21%, with a plateauing survival curve thereafter.^27^ Since CTLA‐4 suppressed excessive immune responses, including those to self‐antigens, anti‐CTLA‐4 Abs can cause a variety of autoimmune reactions, such as autoimmune colitis, hepatitis, thyroiditis, hypophysitis, and interstitial pneumonia.^2^, ^3^ These are referred to as immune‐related adverse events (irAEs), many of which can be controlled with corticosteroids or immunosuppressants, but may require lifelong replacement therapy.

On the other hand, PD‐1 was discovered in 1992 as a gene whose expression is enhanced when T cells induce cell death,^28^ but studies on knockout mice revealed that it is a molecule that suppresses immune reactions.^29^ Some malignant tumors express the ligands PD‐L1 and PD‐L2, and achieve immune escape by transmitting suppressive signals to activated T cells via PD‐1. Anti‐PD‐1 Abs maintain CTL activation by blocking these suppressive signals.^30^ In July 2014, nivolumab was approved in Japan as the world's first anti‐PD‐1 Ab, followed 2 months later by pembrolizumab in the USA. Nivolumab and pembrolizumab are considered equally effective, with ORRs of 42%–45% for nivolumab (CheckMate 066^31^ and 067^32^) and 46% for pembrolizumab (KEYNOTE‐006^33^) in first‐line treatment, and 27% for nivolumab (CheckMate 037^34^) and 22%–34% for pembrolizumab in second‐line treatment and higher (KEYNOTE‐002^35^ and 006^33^). Long‐term survival was also observed with anti‐PD‐1 Abs as well as ipilimumab, with 6.5‐year OS of 42% reported with nivolumab in first‐line treatment^36^ and 7‐year OS of 37.8% reported with pembrolizumab in first‐line treatment.^37^ Since anti‐PD‐1 Abs are significantly more effective and have a lower incidence of serious irAEs than ipilimumab,^31^, ^32^, ^33^ anti‐PD‐1 Abs are now the mainstay of advanced melanoma treatment in guidelines.^38^, ^39^

Since the anti‐CTLA‐4 Ab acts in the priming phase and the anti‐PD‐1 Ab acts in the effector phase, the combination of these two was expected to enhance efficacy. A phase 1 dose‐escalation trial was initiated in 2010 (CA209‐004), which resulted in the use of 1 mg of nivolumab per kg and 3 mg of ipilimumab per kg in combination.^40^ Based on the results of this phase 1 study, a phase 2 RCT (CheckMate 069) comparing nivolumab (1 mg/kg) plus ipilimumab (3 mg/kg) for four doses followed by nivolumab (3 mg/kg) (niv/ipi) with placebo plus ipilimumab (3 mg/kg) for four doses followed by placebo (placebo/ipi) in treatment‐naive, BRAF wild‐type advanced melanoma was conducted.^41^ The primary endpoint of ORR was 61% for niv/ipi and 11% for placebo/ipi, whereas severe AEs were observed in 54% of niv/ipi and 24% of placebo/ipi patients. This led to the approval of nivolumab plus ipilimumab combination therapy for BRAF wild‐type melanoma in the USA in September 2015, which was later extended to BRAF‐mutated melanoma. CheckMate 069 was followed by a phase 3 trial (CheckMate 067) comparing niv/ipi or nivolumab monotherapy with ipilimumab monotherapy in previously untreated advanced melanoma.^32^, ^36^ Co‐primary endpoints were progression‐free survival (PFS) and OS, and median PFS was 11.5, 6.9, and 2.9 months in the niv/ipi, nivolumab, and ipilimumab groups, respectively. Meanwhile, median OS was 72.1, 36.9, and 19.9 months, respectively. Niv/ipi and nivolumab were significantly more effective than ipilimumab, and although the study design did not provide sufficient power to identify a significant difference between niv/ipi and nivolumab, it is recognized that niv/ipi is more efficacious than nivolumab. Meanwhile, because the incidence of serious irAEs is also very high (niv/ipi 59%, nivolumab 21%, ipilimumab 28%), niv/ipi should be considered a treatment for patients with good performance status and in facilities that can handle irAEs. The CheckMate 511 study, conducted as a phase 3b/4 study, compared nivolumab (3 mg/kg) and ipilimumab (1 mg/kg) (niv3/ipi1) with the approved dose of nivolumab (1 mg/kg) and ipilimumab (3 mg/kg) (niv1/ipi3).^42^ Regarding the primary endpoint of safety, 33.9% of niv3/ipi1 and 48.3% of niv1/ipi3 patients had severe AEs, with a significant difference between these groups. ORR was 45.6% and 50.6%, and median PFS was 9.9 and 8.9 months in niv3/ipi1 and niv1/ipi3 patients, respectively, and the clinical effects of these two dosing regimens were considered equivalent. Although it represents off‐label use, niv3/ipi1 is a treatment that would be considered especially in cases with a high risk of irAE. The difference in efficacy between niv/ipi and nivolumab has been shown to vary markedly depending on the presence or absence of BRAF mutations.^43^ In the subgroup analysis at the 6.5‐year follow‐up of CheckMate 067, while there was little difference in efficacy between niv/ipi and nivolumab in BRAF wild‐type patients (6‐year PFS 34% vs 31%, 6.5‐year OS 46% vs 42%), niv/ipi substantially outperformed nivolumab in BRAF‐mutated patients (6‐year PFS 38% vs 23%, 6.5‐year OS 57% vs 43%).^36^ Some reports suggest that niv/ipi is highly effective in BRAF‐mutated melanoma because regulatory T cells, accumulated in the tumor microenvironment, are removed by anti‐CTLA‐4 Ab.^44^, ^45^ However, there are negative reports,^46^ and at this point it remains to be elucidated why niv/ipi is more effective in BRAF‐mutated melanoma.

LAG3 is expressed on activated CTLs and suppresses cellular immunity by binding to MHC class II on APCs.^47^ A phase 2/3 RCT (RELATIVITY‐047) compared anti‐LAG3 Ab relatlimab plus nivolumab combination therapy (rela/niv) with nivolumab monotherapy.^48^ The primary endpoint, median PFS, was 10.2 months for rela/niv and 4.6 months for nivolumab, which were significantly different. Relatlimab plus nivolumab combination therapy was approved in the USA in 2022. Severe AEs were observed in 21.1% of cases for rela/niv and 11.1% for nivolumab. At median follow‐up of 19.3 months, median OS was not reached (NR) for rela/niv (95% confidence interval [CI] 34.2 to NR) and 34.1 months for nivolumab (95% CI 25.2 to NR), showing a tendency for statistical significance (hazard ratio 0.80, 95% CI 0.64 to 1.01, *P* = 0.059).^49^ In a phase 1/2 study (RELATIVITY‐020 part D) of rela/niv in advanced melanoma, two cohorts were established: part D1 allowed only one line of a prior anti‐PD‐1‐containing regimen and part D2 allowed multiple prior lines of anti‐PD‐1/L1‐containing regimens.^50^ The ORR was 12% in D1 and 9.2% in D2, and the median PFS was 2.1 months in D1 and 3.2 months in D2.

## 3 DEVELOPMENT OF SMALL‐MOLECULE MOLECULAR‐TARGETED THERAPY

In 2002, it was reported that BRAF V600E mutation is frequently found in melanoma.^51^ BRAF is a serine/threonine kinase belonging to the RAF kinase family and transduces cell growth stimulation through the RAS‐RAF‐MEK‐ERK signaling pathway.^52^ BRAF with V600E mutation is constitutively active and BRAF has been shown to be one of the important driver genes of melanoma.^51^ The first BRAF inhibitor, vemurafenib, was approved in the USA in 2011 following the results of a phase 3 trial (BRIM‐3) comparing it to dacarbazine in BRAF‐mutated melanoma.^53^ In 2013, the BRAF inhibitor dabrafenib and the MEK inhibitor trametinib were also approved following the results of phase 3 trials (BREAK‐3 for dabrafenib^54^ and METRIC for trametinib^55^). Although they have high response rates of approximately 50%, the median PFS is around 5 months and early resistance occurs. Meanwhile, secondary tumors such as squamous cell carcinoma rarely occur with BRAF inhibitors.^53^, ^54^ Combination therapy with a BRAF inhibitor and a MEK inhibitor (BRAF/MEKi) has been proven to improve ORR, prolong PFS and OS, and reduce the frequency of secondary tumor development compared with a BRAF inhibitor alone.^56^, ^57^, ^58^, ^59^ BRAF/MEKi is now the standard of care for BRAF‐mutated melanoma, and three combinations have been developed based on the results of RCTs (Table 1). In a phase 3 trial (coBRIM) examining the efficacy of vemurafenib plus cobimetinib (vem/cobi), ORR, 5‐year PFS, and 5‐year OS were 70%, 14%, and 31%, respectively.^56^, ^60^ Two phase 3 trials (COMBI‐v and COMBI‐d) investigated the effect of dabrafenib plus trametinib (dab/tra) with an ORR of 64%–68%, and a pooled analysis of COMBI‐d and COMBI‐v showed 5‐year PFS of 19% and 5‐year OS of 34%.^57^, ^58^, ^61^ In the phase 3 COLUMBUS trial, encorafenib plus binimetinib (enco/bini) had an ORR of 64.1%, 5‐year PFS of 22.9%, and 5‐year OS of 34.7%.^59^, ^62^ Adverse events of BRAF/MEKi, such as pyrexia, fatigue, arthralgia, and gastrointestinal symptoms, are common but mostly reversible.^56^, ^57^, ^58^, ^59^ In dab/tra, pyrexia is particularly frequent (about 70%),^57^, ^58^ and in enco/bini, serous retinal detachment is a characteristic adverse event.^59^ The pooled analysis of COMBI‐v and COMBI‐d showed that dab/tra was highly effective in patients with LDH in the normal range and <3 metastatic organs, and the 5‐year OS was very high (70%) in patients who achieved complete response (CR).^61^ The results of coBRIM and COLUMBUS studies were similar, suggesting that BRAF/MEKi alone may be able to maintain long‐term response in selected patients with BRAF‐mutated melanoma.^60^, ^62^

## 4 COMBINATION THERAPY OF ICIS AND BRAF/MEKI IN BRAF‐MUTATED MELANOMA

In expectation of the high response rate of BRAF/MEKi and sustained efficacy of ICIs, a three‐drug combination of an ICI and BRAF/MEKi is being developed for BRAF‐mutated melanoma. In a phase 3 trial (IMspire150) comparing the PD‐L1 Ab atezolizumab plus vem/cobi with vem/cobi, the primary endpoint, median PFS, was 15.1 months in the triplet therapy and 10.6 months in vem/cobi, which were significantly different.^63^ In addition, the triplet regimen atezolizumab plus vem/cobi was approved in the USA in 2020. However, median OS in the second interim analysis was 39.0 months for the triplet therapy and 25.8 months for vem/cobi, which are not significantly different (HR: 0.84, *p* = 0.14).^64^ The National Comprehensive Cancer Network melanoma guidelines do not recommend the triplet regimen as first‐line treatment for BRAF‐mutated melanoma. In addition, a phase 3 study (COMBI‐i) comparing the three‐drug combination of the anti‐PD‐1 Ab spartalizumab plus dab/tra with dab/tra failed to demonstrate a significant improvement of the primary endpoint, PFS.^65^

## 5 SEQUENTIAL THERAPY OF ICIS AND BRAF/MEKI IN BRAF‐MUTATED MELANOMA

In CheckMate 067, about 50% of niv/ipi‐treated patients achieved CR or partial response (PR) at 1 year, and approximately 80% of them maintained their response at 5 years.^36^ In the pooled analysis of COMBI‐v and COMBI‐d, 19% of dab/tra‐treated patients achieved a CR and 71% of them maintained this response at 5 years.^61^ Thus, some patients can be predicted to have a good prognosis with only first‐line treatment, but in most cases sequential treatment is required. RCTs have examined whether BRAF/MEKi or niv/ipi is the best first‐line treatment for BRAF‐mutated melanoma. A phase 3 trial (DREAMseq) in previously untreated BRAF‐mutated melanoma compared niv/ipi as first line and then switched to dab/tra at disease progression with dab/tra as first line and then switched to niv/ipi at disease progression.^65^ The 2‐year OS, the primary endpoint, was 71.8% in patients who started first‐line therapy with niv/ipi, whereas it was 51.5% in those who started first‐line therapy with dab/tra, which differed significantly. The ORR for dab/tra was stable at 43% in the first line and 47.8% in the second line, while the ORR for niv/ipi decreased to 46% in the first line and 29.6% in the second line.

In a phase 2 trial (SECOMBIT) in previously untreated BRAF‐mutated melanoma, patients were randomized to receive niv/ipi as the first line and then switch to enco/bini at disease progression (arm A), enco/bini as the first line and then switch to niv/ipi at disease progression (arm B), or enco/bini and then switch to niv/ipi after 8 weeks of treatment, and enco/bini at disease progression (arm C). The 3‐year OS was 62%, 54%, and 60% in arm A, arm B, and arm C, respectively.^66^ Based on the results of these RCTs, niv/ipi is recommended as first‐line treatment for BRAF‐mutated melanoma.^39^

## 6 DEVELOPMENT STATUS OF MELANOMA TREATMENT IN JAPAN

Table 2 provides a summary of key prospective clinical trials conducted in Japan. A single‐arm phase 2 study (CA184‐202) of ipilimumab (10 mg/kg) plus dacarbazine combination therapy, conducted based on CA184‐024,^3^ was terminated early due to a high incidence of severe hepatitis.^67^ Following the results of a subsequent single‐arm phase 2 study (CA184‐396) with ipilimumab (3 mg/kg) (severe AEs 15%, ORR 10%),^68^ ipilimumab was approved in 2015. However, the number of cases in which ipilimumab was used in first‐line therapy was limited because nivolumab was already approved. A retrospective analysis of the effect of ipilimumab in 61 patients previously treated with anti‐PD‐1 Abs reported a median OS of 7.0 months and an ORR of 4.9%.^69^

**Table 2:** Representative prospective clinical trials in Japan.

Phase	Year	Study	n	Intervention	Line	ORR (%)	Median PFS (months)	Median OS (months)	Any grade AE (%)	Grade ≥3 AE (%)	References
2	2015	CA184‐202	15	Ipilimumab/DTIC	1	13	(−)	(−)	100	73.3	67
2	2015	CA184‐396	20	Ipilimumab	1+	10	2.4	8.7	60	15	68
2	2017	ONO‐4538‐02	35	Nivolumab	2+	28.6	5.5	14.9	85.7	31.4	70
2	2017	ONO‐4538‐08	24	Nivolumab	1	34.8	5.9	32.9	83.3	12.5	71, 72
1b	2017	KEYNOTE‐041	42	Pembrolizumab	1+	24.3	3.9	25.1	81	19	73
2	2018	ONO‐4538‐17	30	niv/ipi	1	43.3	NR	NR	100	76.7	76, 77
1/2	2015	JO28178	11	Vemurafenib	1+	75	13.4	14.3	100	27.3	80
1/2	2018	MEK116885	12	dab/tra	2+	83	(−)	(−)	100	16.7	81

Abbreviations: AE, adverse event; dab/tra, dabrafenib plus trametinib; DTIC, dacarbazine; niv/ipi, nivolumab (1 mg/kg) plus ipilimumab (3 mg/kg) for four doses followed by nivolumab (3 mg/kg); ORR, overall response rate; OS, overall survival; PFS, progression‐free survival.

In a single‐arm phase 2 study (ONO‐4538‐02, *n* = 35) with nivolumab (2 mg/kg, once every three weeks) in the second line or later, ORR was 28.6% and 2‐year OS was 42.9%.^70^ The next single‐arm phase 2 study (ONO‐4538‐08, *n* = 24) with nivolumab (3 mg/kg, once every two weeks) in first‐line therapy showed an ORR of 34.8% and 5‐year OS of 26.1%.^71^, ^72^ In addition, in a single‐arm phase 1b study with pembrolizumab (KEYNOTE‐041, *n* = 42), the ORR was 24.3% and median OS was 25.1 months.^73^ The results of these phase 2 studies of nivolumab and pembrolizumab in Japan tended to show lower effectiveness than those of CheckMate 037,^34^ CheckMate 066,^31^ and KEYNOTE‐006,^33^ which was thought to be because approximately half of the participants in these studies had AM or MM (ONO‐4538‐02 included 26% AM, ONO‐4538‐08 included 29.2% AM and 25% MM, and KEYNOTE‐041 included 28.6% MM and 19% MM).^69^, ^70^, ^71^ The safety profile was similar to that of the corresponding RCTs outside of Japan, with nivolumab approved in July 2014 and pembrolizumab in September 2016. In a prospective observational study of nivolumab (*n* = 124, MM 42, AM 25), the ORR was 17.7% overall, 19% for MM, and 16% for AM, and the median OS was 15.9 months overall, 15.6 months for MM, and 17.5 months for AM.^74^ In the post‐marketing survey of pembrolizumab, the ORR for all 236 patients was 16.5%, 20% for previously untreated (*n* = 145), 11.4% for previously treated (*n* = 88), 17.5% for CM (*n* = 103), 10% for acral (*n* = 60), and 20% for mucosal (*n* = 65).^75^

A single‐arm phase 2 trial (ONO‐4538‐17, *n* = 30) with niv/ipi in the first line showed an ORR of 43.3% and 30‐month OS of 54.2%. This study included eight CM, seven AM, and 12 MM cases, with an ORR of 75% for CM, 42.9% for AM, and 33.3% for MM.^76^, ^77^ The safety profile was like that of CheckMate 067 and niv/ipi was approved in May 2018. In a retrospective observational study with niv/ipi (*n* = 100) at the National Cancer Center Hospital, the ORR for all subjects was 24%, median PFS was 3.3 months, and median OS was 14.5 months. In previously untreated patients (*n* = 48), ORR was 33.3%, median PFS was 6.5 months, and median OS was 25.3 months, and in previously treated patients (*n* = 52), ORR was 15.4%, median PFS was 2.5 months, and median OS was 7.5 months. MM (*n* = 36) had an ORR of 16.7%, median PFS of 3.3 months, and median OS of 14.3 months.^78^ In a retrospective observational study of 111 patients whose first ICI treatment was niv/ipi (prior BRAF/MEKi allowed), the ORR for all subjects was 35% and the 2‐year OS was 50.4%. The 2‐year OS of 33 CM, 28 AM, and 26 MM patients was 45.5%, 63.0%, and 50.0%, respectively.^79^

For targeted therapy, small phase 1 and 2 studies with vemurafenib and dab/tra were conducted and showed efficacy and safety similar to the international phase 3 studies.^80^, ^81^ Vemurafenib was approved in December 2014 and dab/tra in March 2016. Several facilities in Japan participated in the COLUMBUS study,^59^ which led to enco/bini being approved in January 2019. In a retrospective observational study with dab/tra or enco/bini (*n* = 112), the ORR for all subjects was 75%, median PFS was 13.0 months, and median OS was 35 months, comparable to the findings in international phase 3 trials. In a subgroup analysis, the ORR of patients treated with prior ICI (*n* = 34) was 73.5% and it was 64.3% for AM/MM (*n* = 14), indicating a high response regardless of treatment status or melanoma subtype.^82^

Although it appears that there are no ethnic differences in the efficacy of BRAF/MEKi, the efficacy of ICIs in Japan tends to be lower than that in phase 3 trials in the USA and Europe, where most participants are Caucasian. This has been attributed to the high prevalence of AM and MM in Japan, which are less responsive to ICIs. However, recent reports suggest that even CM does not have a higher TMB in East Asians, indicating that ICIs are less effective in East Asians than in Caucasians.^83^ An international retrospective observational study with patients from the USA, Australia, and China analyzed the effect of anti‐PD‐1 Abs by ethnicity and melanoma subgroup.^84^ The 1135 patients included 814 Caucasians, 300 East Asians, 13 Hispanics, and eight Africans, with analyses conducted using the following two groups: Caucasians (*n* = 814) and East Asians/Hispanics/Africans (*n* = 321). Regarding melanoma subtypes, CM and unknown primary (UP) were placed in the same group (*n* = 849, including 710 Caucasians and 139 East Asians and others) and AM, MM, and uveal melanoma (UM) were placed in another group (*n* = 286, including 104 Caucasians and 182 East Asians and others). The differences between the two groups were analyzed. As for background differences, 37% of Caucasians and 21% of East Asians and others were BRAF‐mutated, whereas 49% of Caucasians and 25% of East Asians and others were previously untreated. Overall, the ORR for Caucasians was 49% and median PFS was 9.8 months, while the ORR for East Asians and others was 17% and median PFS was 4.1 months. In the CM/UP cohort, the ORR and median PFS for Caucasians (54%, 14.2 months) were significantly better than for East Asians and others (20%, 5.4 months). In the AM/MM/UM cohort, on the other hand, the ORR for Caucasians was 18% and median PFS was 3.0 months, while the ORR for East Asians and others was 15% and median PFS was 3.6 months. This showed no difference in the effect of anti‐PD‐1 Abs between Caucasians and East Asians and others. The reason why the efficacy of anti‐PD‐1 Abs in CM/UP is higher in Caucasians than in East Asians and others, whereas no ethnic differences in efficacy were observed in AM/MM/UM, is that Caucasians are more UV‐sensitive and may have a higher UV‐induced TMB.

In a retrospective observational study of 336 BRAF‐mutated melanomas in Japan, 236 patients were treated with BRAF/MEKi, 64 with anti‐PD‐1 Ab, and 36 with niv/ipi as first‐line therapy.^85^ With a median follow‐up of 19.9 months, ORR was significantly higher for BRAF/MEKi (69% for BRAF/MEKi, 27% for anti‐PD‐1 Ab, and 28% for niv/ipi); median PFS was also significantly prolonged for BRAF/MEKi (14.7 months for BRAF/MEKi, 5.4 months for anti‐PD‐1 Ab, and 5.8 months for niv/ipi). Meanwhile, median OS was 34.6 months for BRAF/MEKi, 37.0 months for anti‐PD‐1 Ab, and not reached for niv/ipi, with no significant difference among the groups. The ORR for second‐line treatment was unchanged from that of first‐line treatment for BRAF/MEKi (64%), but decreased for ICI (15% for anti‐PD‐1 Ab, 17% for niv/ipi). Comparison of first‐line treatment groups of BRAF/MEKi and niv/ipi by propensity score matching showed no difference in PFS (HR 1.78 for niv/ipi, *p* = 0.15) and in OS (HR 1.003, *p* = 0.95). Based on the results of DREAMseq and SECOMBIT, ICIs (especially nib/ipi) are recommended as first‐line therapy for BRAF‐mutated melanoma.^39^ However, it is unclear whether niv/ipi is optimal as first‐line therapy for BRAF‐mutated melanoma in East Asians, including Japanese, as ICIs may be less effective even in cutaneous melanoma.

## 7 TREATMENT OF AM AND MM

Owing to less UV exposure, the TMB is lower in AM and MM than in CM,^5^, ^6^ and ICIs are thus considered less effective in AM and MM than in CM. Nevertheless, the frequency of BRAF mutations in AM and MM is much lower than in CM,^20^, ^86^ forcing most cases to rely on ICIs for treatment. Few prospective clinical trials have been conducted exclusively on MM and AM because these conditions are rare in Caucasians, accounting for 1%–2% of all melanomas.^13^, ^87^ In addition, although the frequency is high in East Asians, the absolute number of melanomas is small.^14^, ^15^

In a single‐arm phase 2 trial (CheckMate 172) with nivolumab in ipilimumab‐treated advanced melanoma including 723 CM, 63 MM, and 55 AM cases among 1008 participants, the median OS was 25.3 months for CM, 11.5 months for MM, and 25.8 months for AM at a minimum follow‐up of 18 months. There was no significant difference between CM and AM, but the OS for MM was significantly shorter.^88^ A single‐arm, phase 3b study (CheckMate 401) with niv/ipi in previously untreated advanced melanoma included 533 participants, of whom 365 had CM, 32 MM, and 10 AM.^89^ At a minimum follow‐up of 27.3 months, the median OS was not reached for CM, 12.6 months for MM, and 20.8 months for AM, with inferior efficacy in MM and AM. A pooled analysis of KEYNOTE‐001, 002, and 006 (*n* = 1567) was also performed, which included 84 cases of MM with an ORR of 19%, median PFS of 2.8 months, and median OS of 11.3 months. In 1483 patients, excluding MM from the total, ORR was 33%, median PFS was 4.2 months, and median OS was 23.5 months.^90^ Moreover, in a pooled analysis of six prospective clinical trials, including CheckMate 037, 066, and 067, the efficacy of nivolumab monotherapy (MM 86, CM 665), niv/ipi (MM 35, CM 269), and ipilimumab monotherapy (MM 36, CM 269) was analyzed.^91^ In the analysis of MM, ORR was 23.3%, 37.1%, and 8.3% for nivolumab, niv/ipi, and ipilimumab, and median PFS was 3 months, 5.9, and 2.7 months, respectively. In cutaneous melanoma, ORR was 40.9%, 60.4%, and 21.2% for nivolumab, niv/ipi, and ipilimumab, and median PFS was 6.2, 11.7, and 3.9 months, respectively. For all treatments, the effect on MM was lower than that on CM, but even for MM, the efficacy of niv/ipi exceeded that of nivolumab alone.

A retrospective observational study of 329 patients with MM in Japan compared the efficacy of anti‐PD‐1 Ab (*n* = 263) with that of niv/ipi (*n* = 66) and showed no difference between them (ORR 26% vs 29%, median PFS 5.9 vs 6.8 months, median OS 20.4 vs 20.1 months).^92^ In addition, an international, retrospective, observational study compared the efficacy of anti‐PD‐1 Ab and niv/ipi in 545 cases of MM (331 Caucasians, 176 East Asians, 20 others).^93^ Overall, 348 patients were treated with anti‐PD‐1 Ab and 197 with niv/ipi, with no difference in efficacy between them in the overall analysis (ORR 29% vs 31%, median PFS 5 vs 4 months, median OS 19 vs 21 months). Comparison of ORR between Caucasians and East Asians showed no difference in efficacy: 31% for Caucasians and 26% for East Asians for anti‐PD‐1 Ab, and 31% for Caucasians and 33% for East Asians for niv/ipi.

In a retrospective observational study of 193 patients with AM (palm/sole 123, nail 70) treated with anti‐PD‐1 Ab in Japan, ORR was 16%, median PFS was 3.5 months, and median OS was 18.2 months. In comparison with cases in which the palms/soles and nails were affected, ORR was significantly lower in cases involving the nails (8% vs 21%), and median OS was significantly shorter in cases involving the nails (12.8 vs 22.3 months).^94^ In a retrospective observational study of 254 patients with AM in Japan, 209 were treated with anti‐PD‐1 Ab and 45 with niv/ipi.^95^ ORR was significantly higher with niv/ipi than with anti‐PD‐1 Ab (40% vs 16%). Median PFS was 6.6 months for niv/ipi and 4.7 months for anti‐PD‐1 Ab, and median OS was 43.6 months for niv/ipi and 20.7 months for anti‐PD‐1 Ab, with neither of these differences being significant. In an international retrospective observational study of 325 patients with AM (234 plantar, 69 subungual, and 22 palmar), 76% were Caucasian, 184 were treated with anti‐PD‐1 Ab, 59 with niv/ipi, and 82 with ipilimumab.^96^ ORR was 43% for niv/ipi, 26% for anti‐PD‐1 Ab, and 15% for ipilimumab, with the rate for niv/ipi being significantly higher. Median PFS was 5.4 months for niv/ipi, 4.1 months for anti‐PD‐1 Ab, and 3.5 months for ipilimumab, with no significant difference between niv/ipi and anti‐PD‐1 Ab in this regard. Meanwhile, median OS did not differ significantly among the groups (niv/ipi 1.3 years, anti‐PD‐1 Ab 1.9 years, ipilimumab 1.9 years). There was also no difference in efficacy among plantar, subungual, and palmar groups. The efficacy of ICIs for MM and AM appears to be inferior to that for CM. Furthermore, in MM and AM, there may be no advantage of niv/ipi over anti‐PD‐1 Ab monotherapy, or if there is, it seems not to be as obvious as in the case of CM in Caucasian patients.

## 8 CONCLUSION

Since AM and MM have low TMB, the effects of ICIs on them are considered to be inferior to those on CM. This indicates the need for the further development of treatment options in East Asia, including Japan, where the frequency of AM and MM is high. No previous clinical trials with small‐molecule molecular‐targeted therapies for KIT and NRAS have demonstrated an advantage regarding OS, and combination therapies of ICIs with small‐molecule molecular‐targeted drugs are also being considered. A phase 1b trial conducted in China on patients with MM evaluated the efficacy of toripalimab, an anti‐PD‐1 Ab, in combination with axitinib, a multi‐kinase inhibitor. This achieved a favorable ORR of 48.3%,^97^ but median PFS was 7.5 months and median OS was 20.7 months.^98^ Although validation in RCTs is needed, the advantage of toripalimab and axitinib over anti‐PD‐1 Ab monotherapy may not be large. A phase 2 trial is underway in Japan to evaluate the efficacy of imatinib in combination with pembrolizumab in patients with KIT‐mutated melanoma, and the results are awaited.^99^ On the other hand, it has been pointed out that ICIs may not be as effective in East Asians as in Caucasians, even in CM, and a report from China showed no difference in TMB among CM, AM, and MM.^83^ It is necessary to conduct a comparative validation of TMB for CM by ethnicity and, based on the results, prospective trials to examine the efficacy of ICIs by ethnicity would be needed. It would be difficult to extrapolate the results of Caucasian‐dominated RCTs to the treatment of melanoma in East Asia, and evidence from RCTs of melanoma in East Asians is needed.

## CONFLICT OF INTEREST STATEMENT

Authors declare no conflict of interests for this article.

## References

[1] Uchi H , Stan R , Turk MJ , Engelhorn ME , Rizzuto GA , Goldberg SM , et al. Unraveling the relationship between cancer immunity and autoimmunity: lessons learned from models of melanoma and vitiligo. Adv Immunol. 2006;90:215–241. 16730265 10.1016/S0065-2776(06)90006-6

[2] Hodi FS , O'Day SJ , McDermott DF , Weber RW , Sosman JA , Haanen JB , et al. Improved survival with ipilimumab in patients with metastatic melanoma. N Engl J Med. 2010;363:711–723. 20525992 10.1056/NEJMoa1003466 PMC3549297

[3] Robert C , Thomas L , Bondarenko I , O'Day S , Weber J , Garbe C , et al. Ipilimumab plus dacarbazine for previously untreated metastatic melanoma. N Engl J Med. 2011;364:2517–2526. 21639810 10.1056/NEJMoa1104621

[4] Alturki NA . Review of the immune checkpoint inhibitors in the context of cancer treatment. J Clin Med. 2023;12:4301. 37445336 10.3390/jcm12134301 PMC10342855

[5] Alexandrov LB , Nik‐Zainal S , Wedge DC , Aparicio SA , Behjati S , Biankin AV , et al. Signatures of mutational processes in human cancer. Nature. 2013;500:415–421. 23945592 10.1038/nature12477 PMC3776390

[6] Chalmers ZR , Connelly CF , Fabrizio D , Gay L , Ali SM , Ennis R , et al. Analysis of 100,000 human cancer genomes reveals the landscape of tumor mutational burden. Genome Med. 2017;9:34. 28420421 10.1186/s13073-017-0424-2 PMC5395719

[7] Dousset L , Poizeau F , Robert C , Mansard S , Mortier L , Caumont C , et al. Positive association between location of melanoma, ultraviolet signature, tumor mutational burden, and response to anti‐PD‐1 therapy. JCO Precis Oncologia. 2021;5:PO.21.00084. 10.1200/PO.21.00084 PMC8691497 34950838

[8] Marabelle A , Fakih M , Lopez J , Shah M , Shapira‐Frommer R , Nakagawa K , et al. Association of tumour mutational burden with outcomes in patients with advanced solid tumours treated with pembrolizumab: prospective biomarker analysis of the multicohort, open‐label, phase 2 KEYNOTE‐158 study. Lancet Oncol. 2020;21:1353–1365. 32919526 10.1016/S1470-2045(20)30445-9

[9] Hodi FS , Wolchok JD , Schadendorf D , Larkin J , Long GV , Qian X , et al. TMB and inflammatory gene expression associated with clinical outcomes following immunotherapy in advanced melanoma. Cancer Immunol Res. 2021;9:1202–1213. 34389558 10.1158/2326-6066.CIR-20-0983 PMC9414280

[10] Gogas H , Dréno B , Larkin J , Demidov L , Stroyakovskiy D , Eroglu Z , et al. Cobimetinib plus atezolizumab in BRAF^V600^ wild‐type melanoma: primary results from the randomized phase III IMspire170 study. Ann Oncol. 2021;32:384–394. 33309774 10.1016/j.annonc.2020.12.004

[11] Elder DE , Bastian BC , Cree IA , Massi D , Scolyer RA . The 2018 World Health Organization classification of cutaneous, mucosal, and uveal melanoma: detailed analysis of 9 distinct subtypes defined by their evolutionary pathway. Arch Pathol Lab Med. 2020;144:500–522. 32057276 10.5858/arpa.2019-0561-RA

[12] Dimitriou F , Krattinger R , Ramelyte E , Barysch MJ , Micaletto S , Dummer R , et al. The world of melanoma: epidemiologic, genetic, and anatomic differences of melanoma across the globe. Curr Oncol Rep. 2018;20:87. 30250984 10.1007/s11912-018-0732-8

[13] Bradford PT , Goldstein AM , McMaster ML , Tucker MA . Acral lentiginous melanoma, incidence and survival patterns in the United States, 1986‐2005. Arch Dermatol. 2009;145:427–434. 19380664 10.1001/archdermatol.2008.609 PMC2735055

[14] Fujisawa Y , Yoshikawa S , Minagawa A , Takenouchi T , Yokota K , Uchi H , et al. Clinical and histopathological characteristics and survival analysis of 4594 Japanese patients with melanoma. Cancer Med. 2019;8:2146–2156. 30932370 10.1002/cam4.2110 PMC6536943

[15] Tomizuka T , Namikawa K , Higashi T . Characteristics of melanoma in Japan: a nationwide registry analysis 2011–2013. Melanoma Res. 2017;27:492–497. 28609317 10.1097/CMR.0000000000000375

[16] Hayward NK , Wilmott JS , Waddell N , Johansson PA , Field MA , Nones K , et al. Whole‐genome landscapes of major melanoma subtypes. Nature. 2017;545:175–180. 28467829 10.1038/nature22071

[17] Newell F , Johansson PA , Wilmott JS , Nones K , Lakis V , Pritchard AL , et al. Comparative genomics provides etiologic and biological insight into melanoma subtypes. Cancer Discov. 2022;12:2856–2879. 36098958 10.1158/2159-8290.CD-22-0603 PMC9716259

[18] Ward JP , Gubin MM , Schreiber RD . The role of neoantigens in naturally occurring and therapeutically induced immune responses to cancer. Adv Immunol. 2016;130:25–74. 26922999 10.1016/bs.ai.2016.01.001 PMC6087548

[19] The Cancer Genome Atlas Network . Genomic classification of cutaneous melanoma. Cell. 2015;161:1681–1696. 26091043 10.1016/j.cell.2015.05.044 PMC4580370

[20] Sakaizawa K , Ashida A , Uchiyama A , Ito T , Fujisawa Y , Ogata D , et al. Clinical characteristics associated with BRAF, NRAS and KIT mutations in Japanese melanoma patients. J Dermatol Sci. 2015;80:33–37. 26282084 10.1016/j.jdermsci.2015.07.012

[21] Newell F , Wilmott JS , Johansson PA , Nones K , Addala V , Mukhopadhyay P , et al. Whole‐genome sequencing of acral melanoma reveals genomic complexity and diversity. Nat Commun. 2020;11:5259. 33067454 10.1038/s41467-020-18988-3 PMC7567804

[22] Newell F , Kong Y , Wilmott JS , Johansson PA , Ferguson PM , Cui C , et al. Whole‐genome landscape of mucosal melanoma reveals diverse drivers and therapeutic targets. Nat Commun. 2019;10:3163. 31320640 10.1038/s41467-019-11107-x PMC6639323

[23] Dummer R , Schadendorf D , Ascierto PA , Arance A , Dutriaux C , Di Giacomo AM , et al. Binimetinib versus dacarbazine in patients with advanced NRAS‐mutant melanoma (NEMO): a multicentre, open‐label, randomised, phase 3 trial. Lancet Oncol. 2017;18:435–445. 28284557 10.1016/S1470-2045(17)30180-8

[24] Carvajal RD , Antonescu CR , Wolchok JD , Chapman PB , Roman RA , Teitcher J , et al. KIT as a therapeutic target in metastatic melanoma. JAMA. 2011;305:2327–2334. 21642685 10.1001/jama.2011.746 PMC3986039

[25] Graziania G , Tentoria L , Navarra P . Ipilimumab: a novel immunostimulatory monoclonal antibody for the treatment of cancer. Pharmacol Res. 2012;65:9–22. 21930211 10.1016/j.phrs.2011.09.002

[26] Phan GQ , Yang JC , Sherry RM , Hwu P , Topalian SL , Schwartzentruber DJ , et al. Cancer regression and autoimmunity induced by cytotoxic T lymphocyte‐associated antigen 4 blockade in patients with metastatic melanoma. Proc Natl Acad Sci USA. 2003;100:8372–8377. 12826605 10.1073/pnas.1533209100 PMC166236

[27] Schadendorf D , Hodi FS , Robert C , Weber JS , Margolin K , Hamid O , et al. Pooled analysis of long‐term survival data from phase II and phase III trials of ipilimumab in unresectable or metastatic melanoma. J Clin Oncol. 2015;33:1889–1894. 25667295 10.1200/JCO.2014.56.2736 PMC5089162

[28] Ishida Y , Agata Y , Shibahara K , Honjo T . Induced expression of PD‐1, a novel member of the immunoglobulin gene superfamily, upon programmed cell death. EMBO J. 1992;11:3887–3895. 1396582 10.1002/j.1460-2075.1992.tb05481.x PMC556898

[29] Nishimura H , Okazaki T , Tanaka Y , Nakatani K , Hara M , Matsumori A , et al. Autoimmune dilated cardiomyopathy in PD‐1 receptor‐deficient mice. Sience. 2001;291:319–322. 10.1126/science.291.5502.319 11209085

[30] Iwai Y , Ishida M , Tanaka Y , Okazaki T , Honjo T , Minato N . Involvement of PD‐L1 on tumor cells in the escape from host immune system and tumor immunotherapy by PD‐L1 blockade. Proc Natl Acad Sci USA. 2002;99:12293–12297. 12218188 10.1073/pnas.192461099 PMC129438

[31] Robert C , Long GV , Brady B , Dutriaux C , Maio M , Mortier L , et al. Nivolumab in previously untreated melanoma without BRAF mutation. N Engl J Med. 2015;372:320–330. 25399552 10.1056/NEJMoa1412082

[32] Larkin J , Chiarion‐Sileni V , Gonzalez R , Grob JJ , Cowey CL , Lao CD , et al. Combined nivolumab and ipilimumab or monotherapy in untreated melanoma. N Engl J Med. 2015;373:23–34. 26027431 10.1056/NEJMoa1504030 PMC5698905

[33] Robert C , Schachter J , Long GV , Arance A , Grob JJ , Mortier L , et al. Pembrolizumab versus ipilimumab in advanced melanoma. N Engl J Med. 2015;372:2521–2532. 25891173 10.1056/NEJMoa1503093

[34] Weber JS , D'Angelo SP , Minor D , Hodi FS , Gutzmer R , Neyns B , et al. Nivolumab versus chemotherapy in patients with advanced melanoma who progressed after anti‐CTLA‐4 treatment (CheckMate 037): a randomised, controlled, open‐label, phase 3 trial. Lancet Oncol. 2015;16:375–384. 25795410 10.1016/S1470-2045(15)70076-8

[35] Ribas A , Puzanov I , Dummer R , Schadendorf D , Hamid O , Robert C , et al. Pembrolizumab versus investigator‐choice chemotherapy for ipilimumab‐refractory melanoma (KEYNOTE‐002): a randomised, controlled, phase 2 trial. Lancet Oncol. 2015;16:908–918. 26115796 10.1016/S1470-2045(15)00083-2 PMC9004487

[36] Wolchok JD , Chiarion‐Sileni V , Gonzalez R , Grob JJ , Rutkowski P , Lao CD , et al. Long‐term outcomes with nivolumab plus ipilimumab or nivolumab alone versus ipilimumab in patients with advanced melanoma. J Clin Oncol. 2022;40:127–137. 34818112 10.1200/JCO.21.02229 PMC8718224

[37] Robert C , Carlino MS , McNeil C , Ribas A , Grob JJ , Schachter J , et al. Seven‐year follow‐up of the phase III KEYNOTE‐006 study: pembrolizumab versus ipilimumab in advanced melanoma. J Clin Oncol. 2023;41:3998–4003. 37348035 10.1200/JCO.22.01599

[38] Garbe C , Amaral T , Peris K , Hauschild A , Arenberger P , Basset‐Seguin N , et al. European consensus‐based interdisciplinary guideline for melanoma. Part 2: treatment – update 2022. Eur J Cancer. 2022;170:256–284. 35623961 10.1016/j.ejca.2022.04.018

[39] NCCN Clinical Practice Guidelines in Oncology. Melanoma: Cutaneous Version 2.2023. https://www.nccn.org/professionals/physician_gls/pdf/cutaneous_melanoma.pdf

[40] Wolchok JD , Kluger H , Callahan MK , Postow MA , Rizvi NA , Lesokhin AM , et al. Nivolumab plus ipilimumab in advanced melanoma. N Engl J Med. 2013;369:122–133. 23724867 10.1056/NEJMoa1302369 PMC5698004

[41] Postow MA , Chesney J , Pavlick AC , Robert C , Grossmann K , McDermott D , et al. Nivolumab and ipilimumab versus ipilimumab in untreated melanoma. N Engl J Med. 2015;372:2006–2017. 25891304 10.1056/NEJMoa1414428 PMC5744258

[42] Lebbé C , Meyer N , Mortier L , Marquez‐Rodas I , Robert C , Rutkowski P , et al. Evaluation of two dosing regimens for nivolumab in combination with ipilimumab in patients with advanced melanoma: results from the phase IIIb/IV CheckMate 511 trial. J Clin Oncol. 2019;37:867–875. 30811280 10.1200/JCO.18.01998 PMC6455714

[43] van Not OJ , Blokx WAM , van den Eertwegh AJM , de Meza MM , Haanen JB , Blank CU , et al. BRAF and NRAS mutation status and response to checkpoint inhibition in advanced melanoma. JCO Precis Oncol. 2022;6:e2200018. 36130145 10.1200/PO.22.00018

[44] Shabaneh TB , Molodtsov AK , Steinberg SM , Zhang P , Torres GM , Mohamed GA , et al. Oncogenic BRAF^V600E^ governs regulatory T‐cell recruitment during melanoma tumorigenesis. Cancer Res. 2018;78:5038–5049. 30026331 10.1158/0008-5472.CAN-18-0365 PMC6319620

[45] Tang F , Du X , Liu M , Zheng P , Liu Y . Anti‐CTLA‐4 antibodies in cancer immunotherapy: selective depletion of intratumoral regulatory T cells or checkpoint blockade? Cell Biosci. 2018;8:30. 29713453 10.1186/s13578-018-0229-z PMC5907314

[46] Sharma A , Subudhi SK , Blando J , Vence L , Wargo J , Allison JP , et al. Anti‐CTLA‐4 immunotherapy does not deplete FOXP3^+^ regulatory T cells (Tregs) in human cancers. Clin Cancer Res. 2019;25:1233–1238. 30054281 10.1158/1078-0432.CCR-18-0762 PMC6348141

[47] Kreidieh FY , Tawbi HA . The introduction of LAG‐3 checkpoint blockade in melanoma: immunotherapy landscape beyond PD‐1 and CTLA‐4 inhibition. Ther Adv Med Oncol. 2023;15:17588359231186027. 37484526 10.1177/17588359231186027 PMC10357068

[48] Tawbi HA , Schadendorf D , Lipson EJ , Ascierto PA , Matamala L , Castillo Gutiérrez E , et al. Relatlimab and nivolumab versus nivolumab in untreated advanced melanoma. N Engl J Med. 2022;386:24–34. 34986285 10.1056/NEJMoa2109970 PMC9844513

[49] Long GV , Hodi FS , Lipson EJ , Shedendorf D , Ascierto PA , Matamala L , et al. Overall survival and response with nivolumab and relatlimab in advanced melanoma. NEJM Evid. 2023;2. 10.1056/EVIDoa2200239 38320023

[50] Ascierto PA , Lipson EJ , Dummer R , Larkin J , Long GV , Sanborn RE , et al. Nivolumab and relatlimab in patients with advanced melanoma that had progressed on anti‐programmed death‐1/programmed death ligand 1 therapy: results from the phase I/IIa RELATIVITY‐020 trial. J Clin Oncol. 2023;41:2724–2735. 36780608 10.1200/JCO.22.02072 PMC10431305

[51] Davies H , Bignell GR , Cox C , Stephens P , Edkins S , Clegg S . Mutations of the BRAF gene in human cancer. Nature. 2002;417:949–954. 12068308 10.1038/nature00766

[52] Braicu C , Buse M , Busuioc C , Drula R , Gulei D , Raduly L , et al. A comprehensive review on MAPK: a promising therapeutic target in cancer. Cancers (Basel). 2019;11:1618. 31652660 10.3390/cancers11101618 PMC6827047

[53] Chapman PB , Hauschild A , Robert C , Haanen JB , Ascierto P , Larkin J , et al. Improved survival with vemurafenib in melanoma with BRAF V600E mutation. N Engl J Med. 2011;364:2507–2516. 21639808 10.1056/NEJMoa1103782 PMC3549296

[54] Hauschild A , Grob JJ , Demidov LV , Jouary T , Gutzmer R , Millward M , et al. Dabrafenib in BRAF‐mutated metastatic melanoma: a multicentre, open‐label, phase 3 randomised controlled trial. Lancet. 2012;380:358–365. 22735384 10.1016/S0140-6736(12)60868-X

[55] Flaherty KT , Robert C , Hersey P , Nathan P , Garbe C , Milhem M , et al. Improved survival with MEK inhibition in BRAF‐mutated melanoma. N Engl J Med. 2012;367:107–114. 22663011 10.1056/NEJMoa1203421

[56] Larkin J , Ascierto PA , Dréno B , Atkinson V , Liszkay G , Maio M , et al. Combined vemurafenib and cobimetinib in BRAF‐mutated melanoma. N Engl J Med. 2014;371:1867–1876. 25265494 10.1056/NEJMoa1408868

[57] Long GV , Stroyakovskiy D , Gogas H , Levchenko E , de Braud F , Larkin J , et al. Combined BRAF and MEK inhibition versus BRAF inhibition alone in melanoma. N Engl J Med. 2014;371:1877–1888. 25265492 10.1056/NEJMoa1406037

[58] Robert C , Karaszewska B , Schachter J , Rutkowski P , Mackiewicz A , Stroiakovski D , et al. Improved overall survival in melanoma with combined dabrafenib and trametinib. N Engl J Med. 2015;372:30–39. 25399551 10.1056/NEJMoa1412690

[59] Dummer R , Ascierto PA , Gogas HJ , Arance A , Mandala M , Liszkay G , et al. Encorafenib plus binimetinib versus vemurafenib or encorafenib in patients with BRAF‐mutant melanoma (COLUMBUS): a multicentre, open‐label, randomised phase 3 trial. Lancet Oncol. 2018;19:603–615. 29573941 10.1016/S1470-2045(18)30142-6

[60] Ascierto PA , Dréno B , Larkin J , Ribas A , Liszkay G , Maio M , et al. 5‐year outcomes with cobimetinib plus vemurafenib in BRAFV600 mutation‐positive advanced melanoma: extended follow‐up of the coBRIM study. Clin Cancer Res. 2021;27:5225–5235. 34158360 10.1158/1078-0432.CCR-21-0809 PMC9401485

[61] Robert C , Grob JJ , Stroyakovskiy D , Karaszewska B , Hauschild A , Levchenko E , et al. Five‐year outcomes with dabrafenib plus trametinib in metastatic melanoma. N Engl J Med. 2019;381:626–636. 31166680 10.1056/NEJMoa1904059

[62] Dummer R , Flaherty KT , Robert C , Arance A , de Groot JWB , Garbe C , et al. COLUMBUS 5‐year update: a randomized, open‐label, phase III trial of Encorafenib plus binimetinib versus vemurafenib or encorafenib in patients with BRAF V600‐mutant melanoma. J Clin Oncol. 2022;40:4178–4188. 35862871 10.1200/JCO.21.02659 PMC9916040

[63] Gutzmer R , Stroyakovskiy D , Gogas H , Robert C , Lewis K , Protsenko S , et al. Atezolizumab, vemurafenib, and cobimetinib as first‐line treatment for unresectable advanced BRAFV600 mutation‐positive melanoma (IMspire150): primary analysis of the randomised, double‐blind, placebo‐controlled, phase 3 trial. Lancet. 2020;395:1835–1844. 32534646 10.1016/S0140-6736(20)30934-X

[64] Ascierto PA , Stroyakovskiy D , Gogas H , Robert C , Lewis K , Protsenko S , et al. Overall survival with first‐line atezolizumab in combination with vemurafenib and cobimetinib in BRAFV600 mutation‐positive advanced melanoma (IMspire150): second interim analysis of a multicentre, randomised, phase 3 study. Lancet Oncol. 2023;24:33–44. 36460017 10.1016/S1470-2045(22)00687-8

[65] Atkins MB , Lee SJ , Chmielowski B , Tarhini AA , Cohen GI , Truong TG , et al. Combination dabrafenib and trametinib versus combination nivolumab and ipilimumab for patients with advanced BRAF‐mutant melanoma: the DREAMseq trial‐ECOG‐ACRIN EA6134. J Clin Oncol. 2023;41:186–197. 36166727 10.1200/JCO.22.01763 PMC9839305

[66] Ascierto PA , Mandalà M , Ferrucci PF , Guidoboni M , Rutkowski P , Ferraresi V , et al. Sequencing of Ipilimumab plus nivolumab and encorafenib plus binimetinib for untreated BRAF‐mutated metastatic melanoma (SECOMBIT): a randomized, three‐arm, open‐label phase II trial. J Clin Oncol. 2023;41:212–221. 36049147 10.1200/JCO.21.02961

[67] Yamazaki N , Uhara H , Fukushima S , Uchi H , Shibagaki N , Kiyohara Y , et al. Phase II study of the immune‐checkpoint inhibitor ipilimumab plus dacarbazine in Japanese patients with previously untreated, unresectable or metastatic melanoma. Cancer Chemother Pharmacol. 2015;76:969–975. 26407818 10.1007/s00280-015-2870-0 PMC4612320

[68] Yamazaki N , Kiyohara Y , Uhara H , Fukushima S , Uchi H , Shibagaki N , et al. Phase II study of ipilimumab monotherapy in Japanese patients with advanced melanoma. Cancer Chemother Pharmacol. 2015;76:997–1004. 26410424 10.1007/s00280-015-2873-x PMC4612321

[69] Tsutsumida A , Fukushima S , Yokota K , Yoshikawa S , Yamasaki O , Tanemura A , et al. Japanese real‐world study of sequential nivolumab and ipilimumab treatment in melanoma. J Dermatol. 2019;46:947–955. 31531895 10.1111/1346-8138.15073

[70] Yamazaki N , Kiyohara Y , Uhara H , Iizuka H , Uehara J , Otsuka F , et al. Cytokine biomarkers to predict antitumor responses to nivolumab suggested in a phase 2 study for advanced melanoma. Cancer Sci. 2017;108:1022–1031. 28266140 10.1111/cas.13226 PMC5448619

[71] Yamazaki N , Kiyohara Y , Uhara H , Uehara J , Fujimoto M , Takenouchi T , et al. Efficacy and safety of nivolumab in Japanese patients with previously untreated advanced melanoma: a phase II study. Cancer Sci. 2017;108:1223–1230. 28342215 10.1111/cas.13241 PMC5480079

[72] Yamazaki N , Kiyohara Y , Uhara H , Uehara J , Fujisawa Y , Takenouchi T , et al. Long‐term follow up of nivolumab in previously untreated Japanese patients with advanced or recurrent malignant melanoma. Cancer Sci. 2019;110:1995–2003. 30959557 10.1111/cas.14015 PMC6549931

[73] Yamazaki N , Takenouchi T , Fujimoto M , Ihn H , Uchi H , Inozume T , et al. Phase 1b study of pembrolizumab (MK‐3475; anti‐PD‐1 monoclonal antibody) in Japanese patients with advanced melanoma (KEYNOTE‐041). Cancer Chemother Pharmacol. 2017;79:651–660. 28283736 10.1007/s00280-016-3237-x PMC5364262

[74] Yamazaki N , Takenouchi T , Nakamura Y , Takahashi A , Namikawa K , Kitano S , et al. Prospective observational study of the efficacy of nivolumab in Japanese patients with advanced melanoma (CREATIVE study). Jpn J Clin Oncol. 2021;51:1232–1241. 34115870 10.1093/jjco/hyab064 PMC8326387

[75] Yamazaki N , Shimizu A , Ozaki M , Hamada M , Takeuchi N , Ito Y , et al. Real‐world safety and effectiveness of pembrolizumab in Japanese patients with radically unresectable melanoma: an all‐case postmarketing surveillance in Japan. J Dermatol. 2022;49:1096–1105. 35896505 10.1111/1346-8138.16518 PMC9796869

[76] Namikawa K , Kiyohara Y , Takenouchi T , Uhara H , Uchi H , Yoshikawa S , et al. Efficacy and safety of nivolumab in combination with ipilimumab in Japanese patients with advanced melanoma: an open‐label, single‐arm, multicentre phase II study. Eur J Cancer. 2018;105:114–126. 30447539 10.1016/j.ejca.2018.09.025

[77] Namikawa K , Kiyohara Y , Takenouchi T , Uhara H , Uchi H , Yoshikawa S , et al. Final analysis of a phase II study of nivolumab in combinationwith ipilimumab for unresectable chemotherapy‐naïve advanced melanoma. J Dermatol. 2020;47:1257–1266. 32812243 10.1111/1346-8138.15514 PMC7693067

[78] Takahashi A , Namikawa K , Ogata D , Jinnai S , Nakano E , Yamazakin N . Updated analysis of nivolumab and ipilimumab combination therapy in Japanese patients with advanced melanoma. J Dermatol. 2023;50:525–535. 36514836 10.1111/1346-8138.16669

[79] Fujisawa Y , Namikawa K , Yoshino K , Kiniwa Y , Ito T , Kato H , et al. Combined use of nivolumab and ipilimumab in Japanese patients with melanoma: a multicentre retrospective study of 111 cases. Br J Dermatol. 2023;189:223–250. 37017027 10.1093/bjd/ljad114

[80] Yamazaki N , Kiyohara Y , Sugaya N , Uhara H . Phase I/II study of vemurafenib in patients with unresectable or recurrent melanoma with BRAF^V600^ mutations. J Dermatol. 2015;42:661–666. 25884515 10.1111/1346-8138.12873

[81] Yamazaki N , Tsutsumida A , Takahashi A , Namikawa K , Yoshikawa S , Fujiwara Y , et al. Phase 1/2 study assessing the safety and efficacy of dabrafenib and trametinib combination therapy in Japanese patients with BRAF V600 mutation‐positive advanced cutaneous melanoma. J Dermatol. 2018;45:397–407. 29399853 10.1111/1346-8138.14210 PMC5947742

[82] Fujisawa Y , Ito T , Kato H , Irie H , Kaji T , Maekawa T , et al. Outcome of combination therapy using BRAF and MEK inhibitors among Asian patients with advanced melanoma: an analysis of 112 cases. Eur J Cancer. 2021;145:210–220. 33503528 10.1016/j.ejca.2020.12.021

[83] Huang F , Li J , Wen X , Zhu B , Liu W , Wang J , et al. Next‐generation sequencing in advanced Chinese melanoma reveals therapeutic targets and prognostic biomarkers for immunotherapy. Sci Rep. 2022;12:9559. 35688842 10.1038/s41598-022-13391-y PMC9187737

[84] Bai X , Shoushtari AN , Betof Warner A , Si L , Tang B , Cui C , et al. Benefit and toxicity of programmed death‐1 blockade vary by ethnicity in patients with advanced melanoma: an international multicentre observational study. Br J Dermatol. 2022;187:401–410. 35293617 10.1111/bjd.21241

[85] Namikawa K , Ito T , Yoshikawa S , Yoshino K , Kiniwa Y , Ohe S , et al. Systemic therapy for Asian patients with advanced BRAF V600‐mutant melanoma in a real‐world setting: a multi‐center retrospective study in Japan (B‐CHECK‐RWD study). Cancer Med. 2023;12:17967–17980. 37584204 10.1002/cam4.6438 PMC10524053

[86] Curtin JA , Fridlyand J , Kageshita T , Patel HN , Busam KJ , Kutzner H , et al. Distinct sets of genetic alterations in melanoma. N Engl J Med. 2005;353:2135–2147. 16291983 10.1056/NEJMoa050092

[87] Bishop KD , Olszewski AJ . Epidemiology and survival outcomes of ocular and mucosal melanomas: a population‐based analysis. Int J Cancer. 2014;134:2961–2971. 24272143 10.1002/ijc.28625

[88] Nathan P , Ascierto PA , Haanen J , Espinosa E , Demidov L , Garbe C , et al. Safety and efficacy of nivolumab in patients with rare melanoma subtypes who progressed on or after ipilimumab treatment: a single‐arm, open‐label, phase II study (CheckMate 172). Eur J Cancer. 2019;119:168–178. 31445199 10.1016/j.ejca.2019.07.010

[89] Dummer R , Corrie P , Gutzmer R , Meniawy TM , Del Vecchio M , Lebbé C , et al. First‐line, fixed‐duration nivolumab plus Ipilimumab followed by nivolumab in clinically diverse patient populations with unresectable stage III or IV melanoma: CheckMate 401. J Clin Oncol. 2023;41:3917–3929. 37307514 10.1200/JCO.22.02199

[90] Hamid O , Robert C , Ribas A , Hodi FS , Walpole E , Daud A , et al. Antitumour activity of pembrolizumab in advanced mucosal melanoma: a post‐hoc analysis of KEYNOTE‐001, 002, 006. Br J Cancer. 2018;119:670–674. 30202085 10.1038/s41416-018-0207-6 PMC6173747

[91] D'Angelo SP , Larkin J , Sosman JA , Lebbé C , Brady B , Neyns B , et al. Efficacy and safety of nivolumab alone or in combination with ipilimumab in patients with mucosal melanoma: a pooled analysis. J Clin Oncol. 2017;35:226–235. 28056206 10.1200/JCO.2016.67.9258 PMC5559888

[92] Nakamura Y , Namikawa K , Yoshikawa S , Kiniwa Y , Maekawa T , Yamasaki O , et al. Anti‐PD‐1 antibody monotherapy versus anti‐PD‐1 plus anti‐CTLA‐4 combination therapy as first‐line immunotherapy in unresectable or metastatic mucosal melanoma: a retrospective, multicenter study of 329 Japanese cases (JMAC study). ESMO Open. 2021;6:100325. 34839104 10.1016/j.esmoop.2021.100325 PMC8633880

[93] Dimitriou F , Namikawa K , Reijers ILM , Buchbinder EI , Soon JA , Zaremba A , et al. Single‐agent anti‐PD‐1 or combined with ipilimumab in patients with mucosal melanoma: an international, retrospective, cohort study. Ann Oncol. 2022;33:968–980. 35716907 10.1016/j.annonc.2022.06.004

[94] Nakamura Y , Namikawa K , Yoshino K , Yoshikawa S , Uchi H , Goto K , et al. Anti‐PD1 checkpoint inhibitor therapy in acral melanoma: a multicenter study of 193 Japanese patients. Ann Oncol. 2020;31:1198–1206. 32522691 10.1016/j.annonc.2020.05.031

[95] Nakamura Y , Namikawa K , Kiniwa Y , Kato H , Yamasaki O , Yoshikawa S , et al. Efficacy comparison between anti‐PD‐1 antibody monotherapy and anti‐PD‐1 plus anti‐CTLA‐4 combination therapy as first‐line immunotherapy for advanced acral melanoma: a retrospective, multicenter study of 254 Japanese patients. Eur J Cancer. 2022;176:78–87. 36194906 10.1016/j.ejca.2022.08.030

[96] Bhave P , Ahmed T , Lo SN , Shoushtari A , Zaremba A , Versluis JM , et al. Efficacy of anti‐PD‐1 and ipilimumab alone or in combination in acral melanoma. J Immunother Cancer. 2022;10:e004668. 35793872 10.1136/jitc-2022-004668 PMC9260790

[97] Sheng X , Yan X , Chi Z , Si L , Cui C , Tang B , et al. Axitinib in combination with toripalimab, a humanized immunoglobulin G4 monoclonal antibody against programmed cell death‐1, in patients with metastatic mucosal melanoma: an open‐label phase IB trial. J Clin Oncol. 2019;37:2987–2999. 31403867 10.1200/JCO.19.00210 PMC6839911

[98] Li S , Wu X , Yan X , Zhou L , Chi Z , Si L , et al. Toripalimab plus axitinib in patients with metastatic mucosal melanoma: 3‐year survival update and biomarker analysis. J Immunother Cancer. 2022;10:e004036. 35193932 10.1136/jitc-2021-004036 PMC9066368

[99] Hirai I , Tanese K , Fukuda K , Fusumae T , Nakamura Y , Sato Y , et al. Imatinib mesylate in combination with pembrolizumab in patients with advanced KIT‐mutant melanoma following progression on standard therapy: a phase I/II trial and study protocol. Medicine (Baltimore). 2021;100:e27832. 34889232 10.1097/MD.0000000000027832 PMC8663894

